# Stochastic Tracking of Infection in a CF Lung

**DOI:** 10.1371/journal.pone.0111245

**Published:** 2014-10-31

**Authors:** Sara Zarei, Ali Mirtar, Forest Rohwer, Peter Salamon

**Affiliations:** 1 Computational Science Research Center, San Diego State University, San Diego, California, United States of America; 2 Electrical and Computer Eng. Dep/University of California San Diego, San Diego, California, United States of America; 3 Department of Biology, San Diego State University, San Diego, California, United States of America; 4 Department of Mathematics and Statistics, San Diego State University, San Diego, California, United States of America; University of Western Australia, Australia

## Abstract

Magnetic Resonance Imaging (MRI) and Computed Tomography (CT) scan are the two ubiquitous imaging sources that physicians use to diagnose patients with Cystic Fibrosis (CF) or any other Chronic Obstructive Pulmonary Disease (COPD). Unfortunately the cost constraints limit the frequent usage of these medical imaging procedures. In addition, even though both CT scan and MRI provide mesoscopic details of a lung, in order to obtain microscopic information a very high resolution is required. Neither MRI nor CT scans provide micro level information about the location of infection in a binary tree structure the binary tree structure of the human lung. In this paper we present an algorithm that enhances the current imaging results by providing estimated micro level information concerning the location of the infection. The estimate is based on a calculation of the distribution of possible mucus blockages consistent with available information using an offline Metropolis-Hastings algorithm in combination with a real-time interpolation scheme. When supplemented with growth rates for the pockets of mucus, the algorithm can also be used to estimate how lung functionality as manifested in spirometric tests will change in patients with CF or COPD.

## Introduction

Patients with chronic obstructive pulmonary disease (COPD) or cystic fibrosis (CF) have chronic lung inflammation which causes airflow limitation and the scarring of lung tissues. Their airways are generally inflamed and produce excess amounts of mucus that impair the flow of air into and out of their lungs.

Our hypothesis is that the scarring, and ultimate remodeling of a CF lung is mostly due to the contact between the lung lining and the mucus. Inflammatory cytokines induce scars in lung tissue [1]. This contact between mucous biofilm and lung tissue facilitates virulent microbes that also play a role in remodeling a CF lung. In comparison with a normal lung, the airway fluids of CF patients contain large amounts of neutrophils as a result of the inflammatory response [2]. Thus our hypothesis is that mucus accumulation is primarily responsible for damage to the lung and therefore tracking its propagation is a crucial task for better diagnosis and treatment.

Spirometry, the measurement of a patient's breathing, is the standard clinical tool for monitoring lung disease. The two most common spirometric indicators are the forced expiratory volume in one second, FEV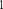, and the forced vital capacity, FVC. FEV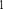 measures the volume of air that can forcibly be blown out in one second while FVC measures the volume of air that can forcibly be blown out after a full inspiration maneuver. Although these tests provide a global measure of airflow obstruction and restriction, they do not give detailed information about the location of mucus blockage. Additional information is available from the repeated imaging of CF patients' lungs. This is usually in the form of chest x-ray, computed tomography (CT) or magnetic resonance imaging (MRI). Despite the fact that CT is an optimal morphological assessment of CF lung changes, the associated exposure to ionizing radiation is a serious obstacle. Therefore, MRI might be the appropriate method for imaging CF patient's lungs [3], [4].

MRI was first introduced as an alternative imaging tool for patients with CF in 1987 [5]. There are numerous methods for the analysis of chest MRI. Theilmann et al. proposed a new MRI imaging method that can spatially locate the pockets of infection and measure the amount of mucus located within each pocket. This information is obtained with a resolution of approximately 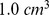 [6]. This resolution is equivalent to the last 10 generations of airways combined. Despite the accuracy level of MRI images, they do not contain any micro-level information on smaller airways. Hence the precise location of infection cannot be obtained from the MRI data.

There have been research studies on mesoscopic modeling of Cystic Fibrosis [7]. Such studies however did not address the micro level information about mucus propagation through the airways. The goal of this research is to provide the clinician with an algorithm that can track the location and propagation of mucus in a CF patient's lungs. Tracking the growth, or shrinkage of these pockets can be correlated to the efficacy of different treatment regimens on each pocket. Assuming further progress in metagenomic and transcriptomic analyses of sputum samples, tracking may allow also correlating the growth or shrinkage of these pockets and the composition of the local microbial community structure within the pocket.

The central tool introduced in the present article for tracking pockets of inflammation is based on using our airflow model [8] in reverse, i.e., as the main ingredient in an inverse problem. Our airflow model can calculate the flow and the total resistance corresponding to a given distribution of mucus obstructions in respiratory airways. The solution of the inverse problem is achieved by randomly sampling the many possible mucus configurations consistent with the current information regarding a patient. Many micro-scale distributions match any observed MRI and spirometric data. The fortuitous finding in the calculations described below is that a large majority of the possible distributions fall within narrow ranges of certain parameters such as which generations of airways contain how much mucus. Can we be certain of these most likely locations being the actual mucus distribution? The fact that most of the distributions consistent with the spirometric and MRI data have these features makes them a good bet while not giving us certainty that any *one* configuration is of this form. In fact, however, repeated MRI measurements separated by small challenges such as coughing or even just taking a few deep breaths reveal that mucus inside the lungs of CF patients show small but discernible movements in response to such challenges. Given this dynamic picture, the possibility that all of the configurations of mucus would avoid the configurations that can be realized the largest number of ways is extremely unlikely. We describe below how to construct such distributions that best represent the state of airways corresponding to the patients lung functionality test values (FEV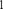 and FVC) and any available MRI or CT data. We use a maximum entropy approach to construct such estimates by sampling all consistent distributions and choosing the one that can be realized the largest number of ways. We find that the predicted microscopic distribution of mucus is generally sharply peaked, allowing us to estimate the distribution of mucus as the one that appears the most frequently in our simulation. The entire computation process can take a long time and would require enormous computational space to run the simulation for the entire 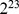 airways in a lung. However after running the simulation once for a patient, we can store most of the data so the next simulations only take a few minutes to complete.

Given the micro-scale distribution obtained from our inverse problem, we can use our physiological model [9] to predict the growth and propagation of this distribution, thereby predicting the progression of the disease. This second model requires a rate for the growth of the mucus volume at each location. Currently only one overall average growth rate is available, a growth rate that was estimated based on a forty year CF population average [9]. Predictions of this model with the average growth rate can nonetheless be compared to actual observed disease progression between exacerbations. For predictions during exacerbations, a database of treatment and community specific growth rates are needed and in-principle available from multiple MRI and metagenomic/transcriptomic analyses. Two such datasets allow the extraction of pocket specific growth rates and corresponding linear extrapolation for the mucus volume in each pocket. This can at least lengthen the times between imaging sessions.

## Methods

### Inverse Problem Using Metropolis-Hastings Algorithm

Our algorithm uses CF patients' spirometry test values of FVC, FEV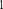 and any available mucus distribution data from CT scans or MRI as its input. It then identifies the distribution of mucus obstructed bronchioles throughout different airway generations. The model assumes the lung airways to be binary branching trees [10] extending over 23 generations from the bronchus down to the alveoli. It further assumes a fractal structure [11]–[13] for the parameters of the binary airway tree.

Considering the fact that there are 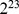 bronchioles in a human lung, there are astronomically many possible configurations for a certain amount of mucus distributed in an airway tree, even given the mucus in each voxel. In fact many of these configurations will result in the same FEV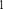 and FVC values. Since exhaustive sampling of these configurations is impossible, we use the Metropolis-Hastings Markov chain Monte Carlo algorithm to sample a few hundred million and base our estimates on such a sample. The goal of this section is to introduce this method.

Our algorithm proceeds from an assumed amount of mucus to be distributed into the 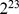 airways. It starts from a random distribution and obtains an unbiased sample of configurations satisfying certain requirements. These requirements are implemented as soft constraints via an energy function 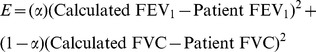
(1)where (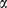) and (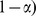 are weight factors. In our algorithm we set 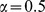. The energy function is used as a way to force the sampling to stay near values with low energy, i.e. configurations with approximately correct spirometric readings.

We then examine successive samples by performing a random walk on the space of mucus configurations. Each move in our random walk reallocates the location of some of the mucus. The resulting distribution of mucus configurations turns out to be sharply peaked in certain natural parameters, a fact exploited by our algorithm.

The Metropolis algorithm is a widely used procedure for sampling a sequence from a specified distribution on a large finite set. It describes equilibrium for systems whose configurations have probability proportional to the Boltzmann factor (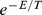). This is a weighting factor which determines the relative probability that the system will be found in a particular configuration at energy 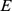 when the temperature of the environment is 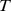 [14]. We used a constant temperature, 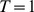, making the probability of a configuration with energy 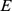 proportional to 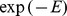.

The following steps describe the Metropolis algorithm [15].

• Initiate the sampling from an arbitrary configuration 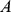, with known energy 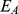.

• Define a new neighbor configuration 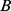 from configuration 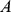.

• Calculate the new configuration energy, 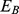. This trial move is then either accepted or rejected according to the following simple probabilistic rule.

• If 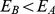 we accept the new configuration.

• if 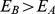, we may accept configuration 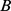 with the following probability.



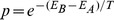
(2)


• Repeat until sufficient number of configurations have been collected.

To apply the Metropolis algorithm to our model, we need to define neighboring configurations. Our definition moves a certain amount of mucus between a few chosen bronchioles. This keeps the energy re-evaluation step computationally cheap and, provided we allow such rearrangement between all types of airways, we avoid the problems created by local minima. This assures that the system settles into and stays near the lowest energy configurations as the simulation proceeds [16].

### Two-Dimensional Probability Density Function Estimation (PDFE-2D) of Mucus Obstructions

Imaging provides us with the data that corresponds to the spatial location of infection pockets and the amount of mucus within each pocket. Both MRI and CT images can have various resolutions and our approach is scalable to any resolution. For concreteness below we work with a resolution of 1 cm^3^ and refer to this smallest volume as a voxel - a volume element.

Since on average the total lung capacity of an adult human is about 6 liters [17], an image will have about 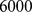 voxels. Using the binary tree structure of the lung airway, we set these voxels at the end of the 13th generation to approximately match the number of these elements to the number of subtrees that remain. Since 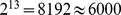, we can identify the mucus in the voxel with the mucus in the subtree of 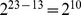 bronchioles terminating in the alveoli. Thus each voxel in our lung model represents a binary tree structure that has a total of 10 bifurcations, from generation 13 to 23, with a known total amount of mucus obtained from imaging data.

Using a three-dimensional model of the human airway tree that was developed by [18], we are able to map each MRI or CT scan voxel into our lung model's voxels with their corresponding mucus content. The main task of our algorithm is to locate the infection in the airway tree structure of a CF lung. To achieve this goal, we use two summary features for each voxel: (1) the percent of alveoli that are accessible, i.e., not totally blocked and (2) the total resistance to flow from the alveoli to the 13th generation brochiole bronchiole assigned to the voxel. These two microscopic features correspond loosely to the spirometric indicators FVC and FEV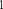, respectively.

In order for us to calculate each voxel's resistance and the percent of accessible alveoli (for simplicity, we refer to this as the AA%), we first have to define how mucus is distributed in a voxel's binary tree structure. There is an astronomical number of configurations for filling a binary tree of 10 generations with a given amount of mucus. Approximately, it is given by 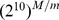 where 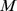 is the total mucus volume and 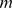 is the volume of the smallest bronchiole. Thus even at this level, we used Metropolis-Hastings to sample many mucus configurations, recording the values of AA% and resistance for each configuration. We set the amount of mucus within each voxel and calculate the distribution of AA% and resistance: our PDFE-2D distribution.

Hence if a voxel contains 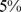 mucus, the first step is randomly filling up the voxel's airway tree with the specified mucus amount. Then using the Metropolis algorithm, at each state we move only a fraction of the mucus within a bronchiole that is equivalent to the smallest bronchiole's volume in a lung airway tree. We refer to this as the “unit volume”. Once a unit volume is moved to a different location, the corresponding voxel's parameters (AA% and resistance) are recalculated.

**Figure 1:**
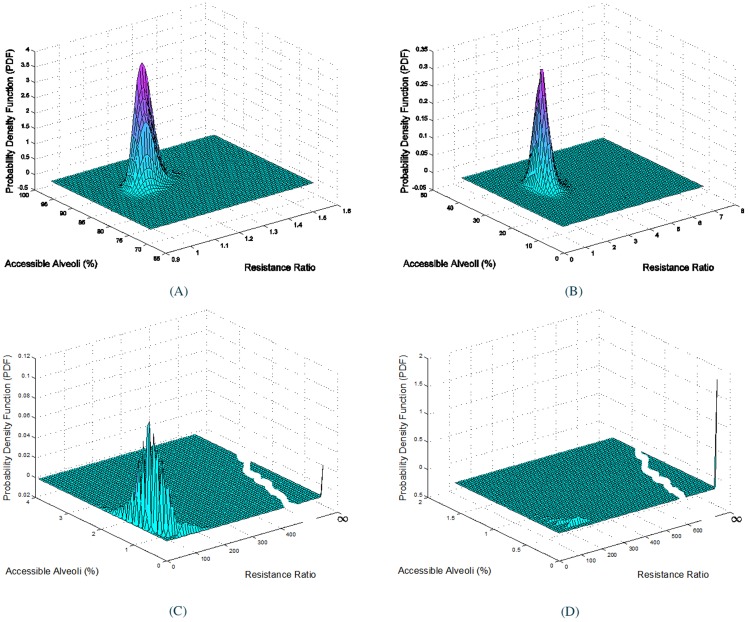
Probability density function for different percent volume of mucus in a small voxel of the lung. **(A)** 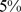, **(B)** 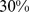, **(C)** 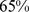 **and (D)** 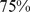 Each voxel represents a subtree from generation 13 to 23 of the binary tree structure of lung. The x-axis is the corresponding airflow resistance and the y-axis shows the percent accessible alveoli.

In order to expedite the computational process; Dulcinea computing clusters from the Computational Science Research Center at San Diego State University were used for collecting almost 54 million samples. The Dulcinea computing clusters contains 12 workstations each with Dual-Quad Xeon central processing unit (CPU) (E5520 2.27GHz) and Dual Tesla graphic processing unit (GPU) (M1060) which provides the total of 96 CPU cores. The cluster system utilizes 3GB of memory per CPU core for nodes 1 to 10 and utilizes 6GB of memory per CPU core for nodes 11 and 12. After obtaining these samples the probability distributions for different amount of mucus are calculated. Figure 1A to Figure 1D illustrate the probability density function for (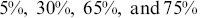) mucus respectively. As shown in Figure 1A, when there is only 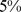 mucus in a voxel, the most likely configuration has 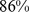 of its alveoli accessible and the voxel's resistance increases by a factor of almost 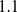. When the mucus level reaches almost 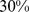 there are only 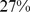 accessible alveoli and the voxel resistance is almost 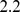 times a healthy voxel with no mucus. On the other hand in Figure 1C and 1D the number of accessible alveoli value approaches zero while the resistance value reaches infinity. This refers to a case that a voxel is almost completely filled with mucus to an extent that no more air can pass through and therefore blocks all the corresponding alveoli at the end of the branching tree.

**Figure 2:**
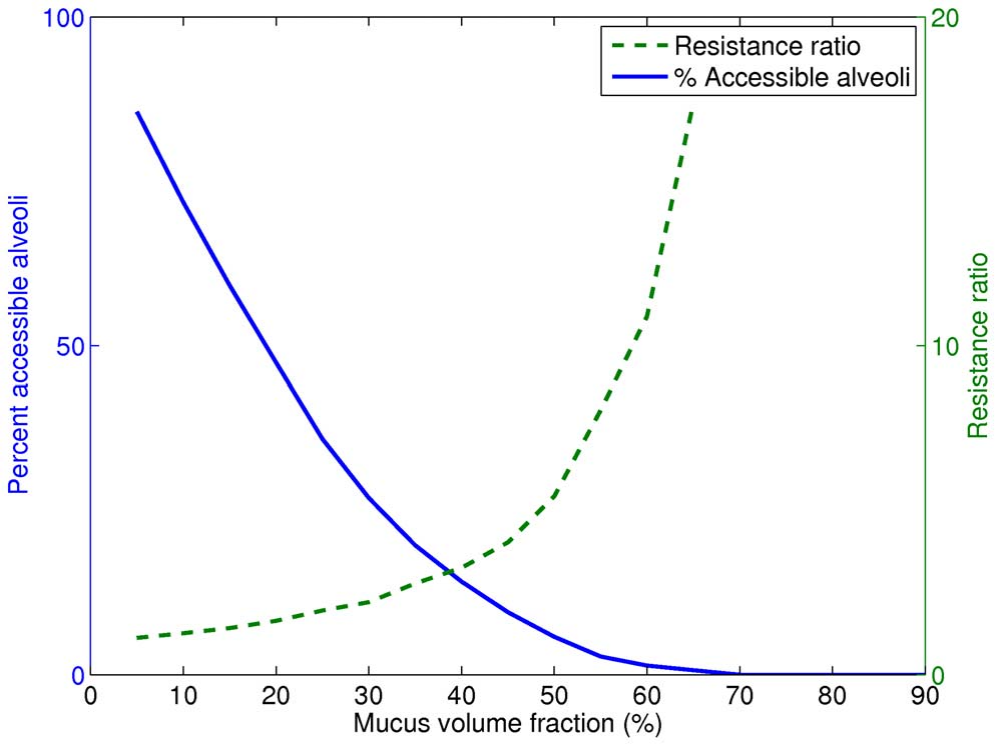
Maximum likelihood of percent of accessible alveoli AA% and airflow resistance for a given mucus volume fraction inside a voxel. The x-axis is mucus volume% and the two y-axes show the most probable combination of the airflow resistance ratio and percent of accessible alveoli. The resistance value increases as the mucus amount rises. Number of accessible alveoli decreases as the mucus volume grows, which leads to a lower lung functionality test value or FVC value.

Figure 2 displays the maximum likelihood combinations of AA% and resistance ratio for different amount of mucus in a voxel. As the mucus reaches almost 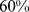 of the available airway volume in the voxel, there is no remaining access to the alveoli and as a result there is no gas exchange taking place in that particular part of the airway tree. After collecting these distributions, the model can initiate the prediction steps as well as providing the microlevel information about the location of obstructed bronchioles. We will discuss each outcome in the next two sections. Please note that all data underlying the findings of this section have been discussed in the manuscript. Other than the massive computing power needed to produce the findings in Figure 1 and Figure 2, all the relevant data have been shared.

## Results

### Micro-Level Information on Obstructed Bronchioles

In order to spatially locate each bronchiole in a human airway tree we used Kitaoka et al.'s three-dimensional model [18]. Once we receive the imaging data for each voxel, we need to map the values to our model's voxel using spatial location coordinates. Next we use the probability density function that we found in the previous section to determine the corresponding voxel parameters, AA% and resistance ratio, sampled according to the PDFE-2D distributions. The model randomly select a combination of percent accessible alveoli and resistance for each voxel in a way that the total resistance and number of accessible alveoli from these voxels provide the same value as the patient's FEV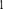 and FVC. There is a complex calculation taking place in parallel to obtain the rate of flow (FEV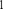) from the total resistance of all the voxels. To find the distribution of voxel parameters we use the Metropolis algorithm to focus the Markov chain to sample many configurations meeting our constraints. This is achieved by choosing the configuration energy provided in Eq(1).

At this stage we have mapped the voxels from imaging data into our model in a way that the total rate of airflow and the accessible alveoli of the lung model resemble the corresponding patient's FEV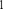 and FVC values respectively. Once the current state of a CF lung is implemented we can obtain clinically useful information, such as mucus distribution within each voxel or predicted future states of each voxel and associated FEV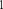 and FVC values as described in the next section.

**Figure 3:**
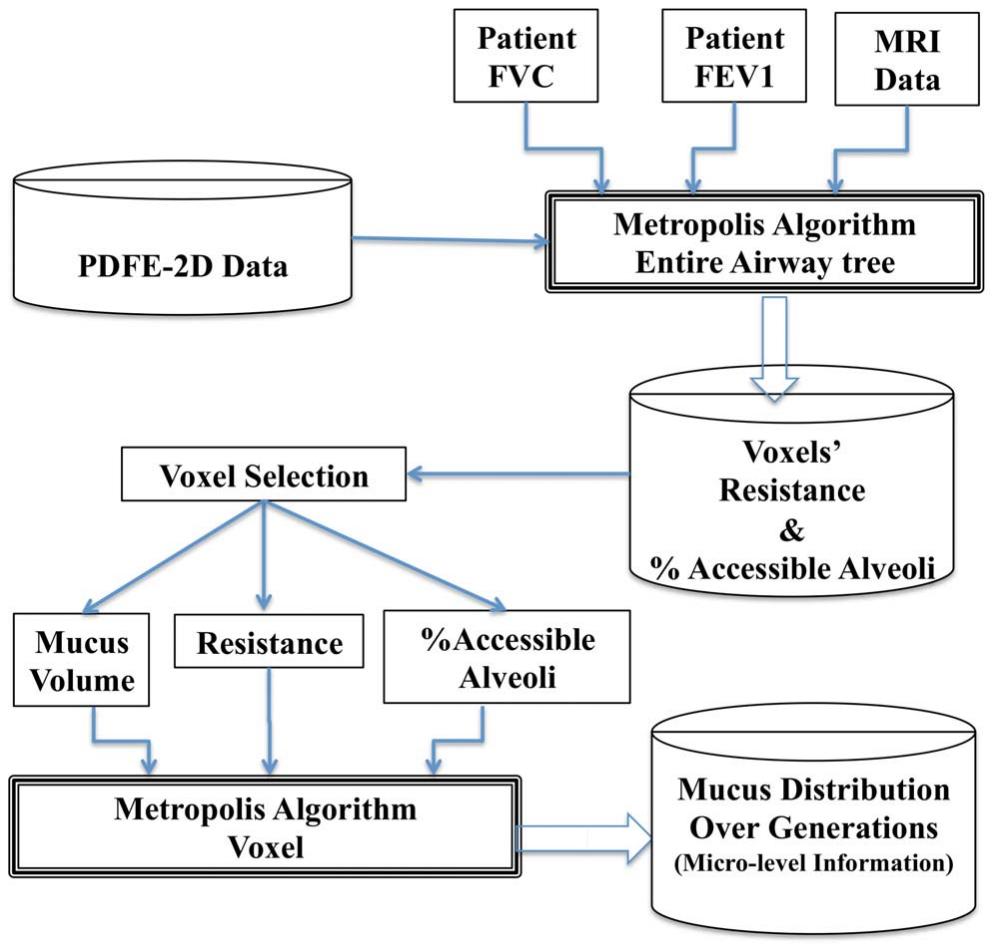
Generating the Micro-level information on obstructed bronchioles. This flowchart shows how the algorithm produces the mucus distribution of a selected voxel. Using the lung functionality test values: FEV1 and FVC and the data obtained from MRI lung imaging we can obtain the corresponding mucus distributions among the different generations of lung.

**Figure 4:**
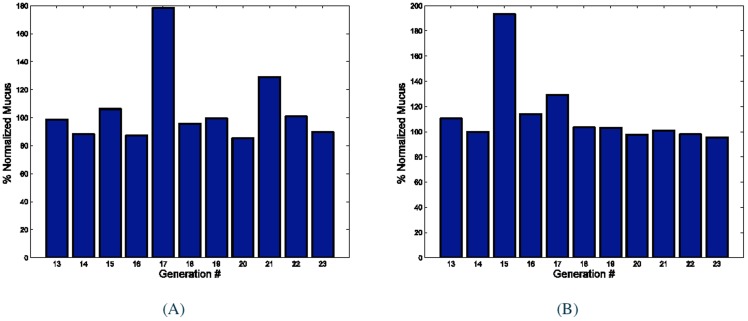
Mucus distribution among different generations of lung for two selected voxels. Figure (A) represents a voxel with 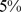 mucus volume that has 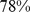 accessible alveoli: AA and resistance ratio of 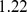. Figure (B) repsresents represents a voxel with 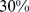 mucus that has 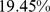 AA and resistance ratio of 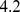. The x-axis is the generation number of the corresponding bronchioles in the selected voxel. The y-axis shows the normalized mucus present, which is equal to the ratio of the mucus amount in a generation to the total mucus volume in a voxel, divided by the ratio of the air volume in that generation to the total voxel volume.

Figure 3 displays the flowchart of this process. As shown in this flowchart, once we have all the voxels' parameters, we can select certain voxels for further analysis. We again apply the Metropolis algorithm on the selected voxel in order to visualize the distribution of mucus in its airway tree structure. For the chosen voxel, we have its mucus volume, its resistance and its AA%. We randomly fill out the voxels' airway tree to reach their corresponding mucus volume. At each iteration the new resistance and AA% are collected. To move to the neighbor configuration we move a unit volume of mucus in a bronchiole to keep the total mucus volume of the voxel fixed. The state energy we use for this step is as follows: 
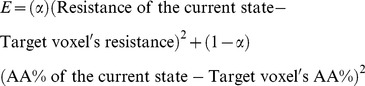
(3)where 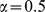. After we obtained enough samples we constructed the corresponding mucus distribution for the selected voxels. Figures 4 displays two examples of the mucus distribution for voxels that contained (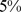) and (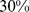) mucus. As shown in these figures, as the percent mucus increases, the dominantly filled generation moves to bigger bronchioles. The y axis repressnts represents % normalized mucus where: 
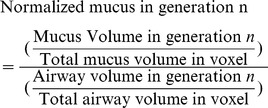
(4)


### Predicting future values of FEV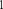 and FVC

**Figure 5:**
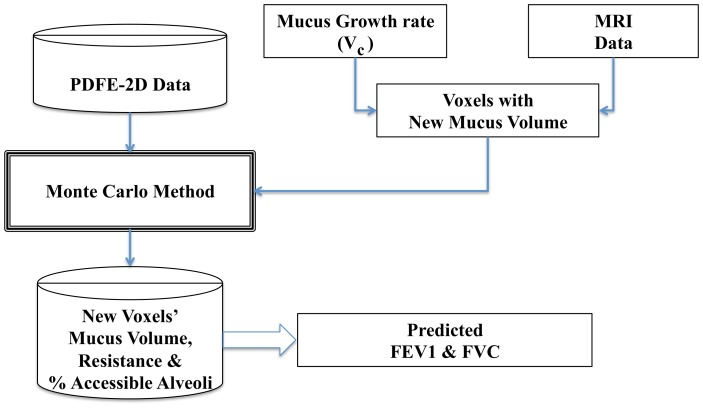
FEV 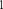 and FVC values predictions predicted from the lung model. This flowchart illustrates the process of how our lung model predicts the FVC and FEV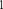 of a CF patient given the previous mucus content of each voxel along with a mucus growth rate.

**Figure 6:**
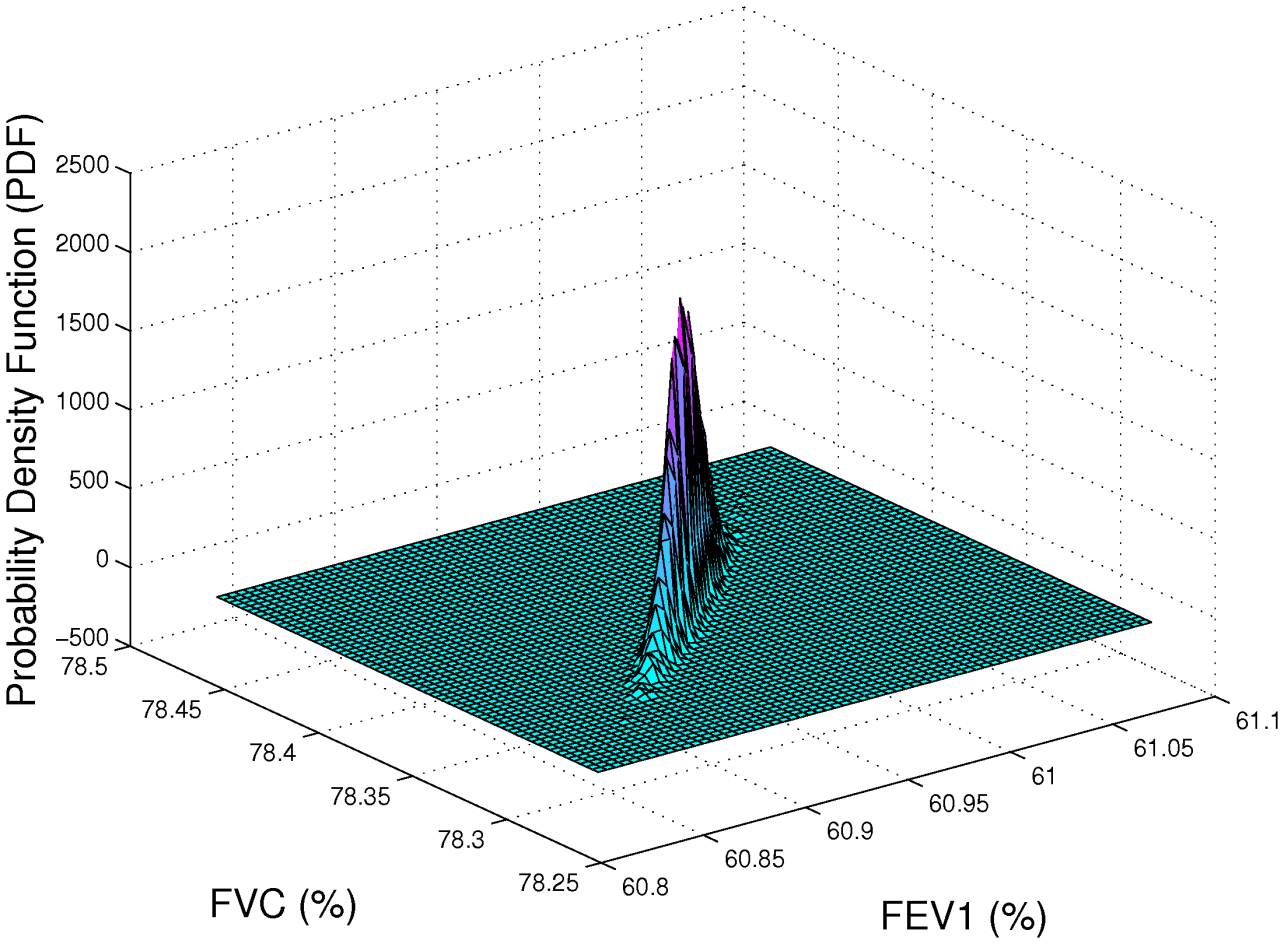
FEV 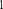 and FVC probability density function estimation. The x-axis shows the predicted FEV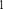 values and the y-axis is the predicted FVC values for a hypothetical the synthetic lung example described in the text.

In this section we use the mucus distribution and growth model presented in [9] to make predictions about the lung functionality of a CF patient. As can be seen in Figure 5 we use the imaging voxels' data and the constant mucus growth rate from [9], or, if available, infection and treatment specific growth rates specific to each voxel to predict the mucus growth in each pocket of infection. The model again resorts to the Monte Carlo method to randomly select the AA% and resistance from our model described in the PDFE-2D section and calculates the new FEV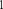 and FVC at each iteration and stores their values. After collecting enough samples we can predict the FEV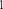 and FVC distributions for a patient after the indicated time period. For example, Figure 6 displays the probability density function of the predicted FEV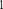 and FVC. As shown in the figure it is predicted that FEV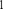 and FVC of our CF patient are approximately 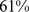 and 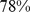 respectively.

**Figure 7:**
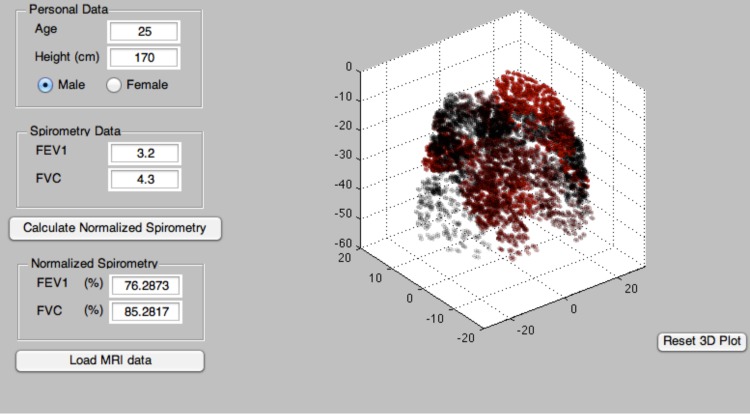
In this window user can enter the input parameters: patient's age, height, sex and Imaging data. The Model displays a 3D-lung that contains all the MRI/CT scan voxels.

**Figure 8:**
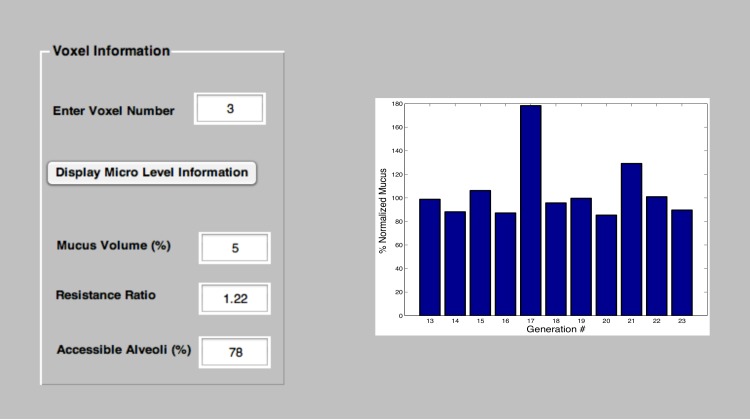
This window shows the mucus distribution among generations. The user selects a voxel and the model displays its corresponding parameters along with the micro-level information.

**Figure 9:**
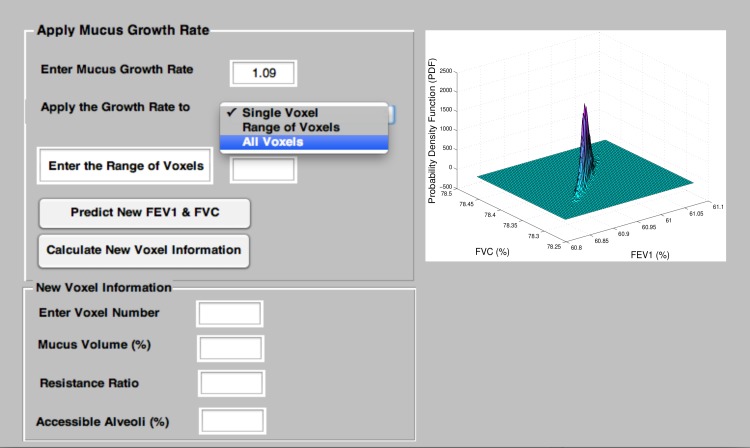
This window provides the user with a probability density function of the predicted FEV 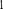 and FVC. User can enter a specific mucus growth rate for different voxels and the new FEV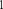 and FVC will be calculated.

This method can even be applied to follow a patient's progress with out fewer imaging tests. Since most CF patients take spirometry test more often than any imaging tests, we can use our model without recourse to imaging data. This time the model takes only the FEV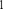 and the FVC values of a patient as input. After applying the Metropolis algorithm (using additional rearrangements of mucus between voxels) the model can still provide an estimate of the distribution of mucus throughout the airway tree. This can be propagated using the constant any hypothetical growth rates to predict the next spirometric test. We have created a Matlab GUI version of the algorithm; its sample output is shown in Figures 7, 8 and 9.

Since the mean mucus growth rate was estimated based on years of data from many CF patients, such predictions should work reasonably well for a patient between exacerbations. Our model can also be used to predict the progression during exacerbations, albeit with voxel specific growth rates informed by much more data than we currently possess. Using one mean mucus growth rate is a shortcoming not of our algorithm but rather of the paucity of data to which our predictions have been applied. There is every indication that soon we will have reasonable metagenomic [19]–[22] and metabolomic [23] tools to assess the microbial composition present in a CF lung and will be able to infer growth rates that are specific to the community composition as well as the antibiotic administered. Entering voxel specific growth rates based on more information than we at present possess and using our program to test predictions can move our understanding of the patient's state to a new and quantitative level.

We can improve the accuracy level of the model by extending the resolution of our PDFE-2D distributions. The current PDFEs were constructed with 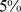 bins as described in PDFE-2D model section.

## Discussion

With current research studies about cystic fibrosis, CF treatment is poised for great strides. Non-genetic treatments such as Ivacaftor (trade name Kalydeco, developed as VX-770) only works on patients with a certain mutation of cystic fibrosis which accounts for 4–5% of cystic fibrosis cases [24].

While an eventual cure for the disease by replacement of the defective gene is likely, we expect such treatment not to be available anytime soon. Rather we anticipate that new observational tools such as metagenomics, transcriptomics, metabolomics and MRI imaging coupled with our modeling approach will give the clinician unprecedented ability to follow and treat the disease. The models will provide quantitative predictions of responses to various drug regimens and prescribe adaptively implemented optimal controls for treatment. Predicting the impact of mucus growth on lung functionality will correlate the current stage of the disease with how infection has been propagating throughout different generations of lung. This will enable the physician with a tool to track this propagation of infection.

The various sub-models required here will soon be informed by data characterizing the microbial communities present. Such data comes from metagenomic and transcriptomics analyses of sputum samples and metabolomic analyses of exhaled air. In our current model we predict the dynamic distribution of mucus in a CF lung in the absence of treatment as a stepping stone for eventually eventual treatment and microbial community specific modeling of the treatment response.

According to a research study done by Willner et al. [25] microbial diversity in a CF lung is much higher than suggested by culturing alone. They were able to characterize the diversity of microbial communities in tissue sections from anatomically distinct regions of the CF lung. Their result indicated that microbial communities in the Cystic Fibrosis lung are spatially heterogeneous.This Spatial heterogeneity will cause regional differences in microbial biomass and antibiotic resistance. The next version of the model, can use their results to adjust the parameters according to the microbial communities found and the treatment administered (e.g., timing of antibiotic administration, types of antibiotics, steroids, etc). The present model should be taken as a proof-of-concept step toward that goal. This will provide an opportunity for the researcher, and eventually the clinician, to access a framework for accurate quantitative predictions.

Our methods are completely scalable – the 1.0 cm^3^ for the size of one voxel was for illustration. Any resolution scale however forces estimation of the distribution on spatial scales below this resolution. Since estimation of the distribution on finer spatial scales would perforce need the solution of an inverse problem with many possible solutions and since the movement of mucus hinted that the “right answer” would in any case not be a unique distribution, we were led naturally to using Monte Carlo estimation methods for the most likely distribution and the corresponding spirometric observables. The resulting models and algorithms form a clinically useful tool with which to reassess the various simplest possible sub-models and assumptions used in our work so far. These models can play a crucial role in future treatments of the disease.

## Supporting Information

File S1 **The supporting file Psudo.pdf contains the psudo code of the algorithms and models mentioned in this manuscript.**
(PDF)
