# Supplementary material for: Stochastic Tracking of Infection in a CF Lung
Source: PLoS One. 2014 Oct 31;9(10):e111245. doi: 10.1371/journal.pone.0111245 (PMC4216002; doi:10.1371/journal.pone.0111245)
Supplement: File S1 — The supporting file Psudo.pdf contains the psudo code of the algorithms and models mentioned in this manuscript. (PDF) [file pone.0111245.s001.pdf]

Pseudo Codes

0.1 Lung Model

```
Consider lung as a full binary tree with N nodes.
P(n) returns parent of node n.
C(n) returns both children of node n.
Cs(n) returns all children of node n to the leaves.
Pc(n) probability of colonization of n.
state(n) state of tube n.
mucus(n) mucus volume in tube n.
mucus_time(n) the first time a tube colonized.
scar(n) amount of scar in tube n.
lambda is the increase in Pc if its neighbors are
   colonized.
alpha is the ratio for trigerring the treatment. 0 <
   alpha < 1.


Lung Simulation:
for t = 1 to simulation_time:
    for n = 1 to N:
        case state(n)
            normal:
                With Pc(n):
                    state(n)=colonized
                    mucus(n)+=Vc
                    mucus_time(n)=t
                    Pc(P(n))+=lambda
                    Pc(C(n))+=lambda
            colonized:
                mucus(n)+=Vc
                if mucus(n)>=tube_vol(n):
```

```
                    state(n)=nonfunctional
                    Pc(P(n))=1.0
                    state(Cs(n))=nonfunctional
                if mucus_time(n)+T_scarring<=t:
                    state(n)=scarred
                    scar(n)+=Rs
            scarred:
                scar(n)+=Rs
                if scar(n)>=scare_threshold:
                    state(n)=dead
                    Pc(P(n))=1.0
                    state(Cs(n))=dead
                mucus(n)+=Vc
                if mucus(n)>=tube_vol(n):
                    state(n)=nonfunctional
                    Pc(P(n))=1.0
                    state(Cs(n))=nonfunctional
    if Vcurrent<alpa*VLast:
        for n = 1 to N:
            case state(n)
                colonized:
                    mucus(n)=0.0
                    state(n)=normal
                scarred:
                    mucus(n)=0.0
                    mucus_time(n)=t
                nonfunctional:
                    mucus(n)=0.0
                    if scar(n)==0.0:
                        state(n)=normal
                    else :
                        state(n)=scarred
                        mucus_time(n)=t
```

0.2 Metropolis-Hastings Algorithm

```
N is number of samples needed.
```

```
General Metropols algorithm:

A=Create_arbitrary_configuration()
for i =1 to N:
    B=Neighbor(A)
    Ea=Energy(A)
    Eb=Energy(B)
    if Eb<Ea:
        A=B
    else:
        with probability exp(Eb-Ea):
            A=B
    keep(A)
```

0.3 Two-Dimensional Probability Estimation of Mucus Obstructions

```
It is based on the general Metropolis algorithm.
P is the percent of lung mucus in a voxel given as input.

Create_arbitrary_configuration:
vol_precision=smallest_tube_leaf
target_vol=P*voxel_volume
current_vol=0.0
while target_vol-current_vol>2*vol_precision :
    if current_vol< taget_vol :
        n=randomly select a tube
        current_vol+=vol_precision
        fill n with vol_precision
    else:
        n=randomly select a filled tube
        current_vol-=vol_precision
        remove from n vol_precision
return configuration
__________

Energy is always zero
Energy(A):
```

```
return 0

————

Neighbor(A):
move_vol=20*vol_precision
semi_target_vol=target_vol-move_vol
while semi_target_vol-current_vol>2*vol_precision :
    if current_vol< taget_vol :
        n=randomly select a tube
        current_vol+=vol_precision
        fill n with vol_precision
    else:
        n=randomly select a filled tube
        current_vol-=vol_precision
        remove from n vol_precision
while target_vol-current_vol>2*vol_precision :
    if current_vol< taget_vol :
        n=randomly select a tube
        current_vol+=vol_precision
        fill n with vol_precision
    else:
        n=randomly select a filled tube
        current_vol-=vol_precision
        remove from n vol_precision
return configuration
```

0.4 Voxel Resistance and Accessible Alveoli

```
It is based on the general Metropolis algorithm.
N is the number of voxels in lung
mucus(n) returns the percent of voxel n filled with mucus
Create_arbitrary_configuration:
for n= 1 to N:
    distribution=select closest PDFE to mucus(n)
    X=randomly select a point in distribution
    AA(n)=AA(X)
    Res(n)=Res(X)
```

```
return configuration

________
alpha is the weighting parameter here it is 0.5
Energy(A):
FEV1perc=calculate FEV1 of configuration A
FVCperc=calculate FVC of configuration A
return alpha*(FEV1perc-patient_FEV1perc)^2+(1-alpha)*(
   FVCperc-patient_FVCperc)^2

________
m voxels will change.
Neighbor(A):
S= a set of m randomly selected voxels out of N
for n in S:
     distribution=select closest PDFE to mucus(n)
     X=randomly select a point in distribution
     AA(n)=AA(X)
     Res(n)=Res(X)
return configuration
```

0.5 Micro-Level Information on Obstructed Bronchioles

```
It is based on the general Metropolis algorithm.
P is the percent of lung mucus in a voxel given as input.

Create_arbitrary_configuration:
vol_precision=smallest_tube_leaf
target_vol=P*voxel_volume
current_vol=0.0
while target_vol-current_vol>2*vol_precision :
     if current_vol< taget_vol :
          n=randomly select a tube
          current_vol+=vol_precision
          fill n with vol_precision
     else:
          n=randomly select a filled tube
          current_vol-=vol_precision
```

```
        remove from n vol_precision
return configuration
————————
alpha is the weighting parameter here it is 0.5
Energy(A):
AAper=calulate AA of configuration A
Res=calculate
return  alpha*(AAper-voxelAAper)^2+(1-alpha)*(Res-
   voxelRes)^2

————————


Neighbor(A):
move_vol=20*vol_precision
semi_target_vol=target_vol-move_vol
while semi_target_vol-current_vol>2*vol_precision :
    if current_vol< taget_vol :
        n=randomly select a tube
        current_vol+=vol_precision
        fill n with vol_precision
    else:
        n=randomly select a filled tube
        current_vol-=vol_precision
        remove from n vol_precision
while target_vol-current_vol>2*vol_precision :
    if current_vol< taget_vol :
        n=randomly select a tube
        current_vol+=vol_precision
        fill n with vol_precision
    else:
        n=randomly select a filled tube
        current_vol-=vol_precision
        remove from n vol_precision
return configuration
```
